# Time course of blood lactate levels, inflammation, and mitochondrial function in experimental sepsis

**DOI:** 10.1186/s13054-017-1691-4

**Published:** 2017-05-12

**Authors:** Thiago Domingos Corrêa, Adriano José Pereira, Sebastian Brandt, Madhusudanarao Vuda, Siamak Djafarzadeh, Jukka Takala, Stephan Mathias Jakob

**Affiliations:** 1 Department of Intensive Care Medicine, Inselspital, Bern University Hospital, University of Bern, Bern, Switzerland; 2 Intensive Care Unit, Hospital Israelita Albert Einstein, Avenida Albert Einstein, 627/701, 5th floor, Sao Paulo, 05651-901 Brazil

**Keywords:** Sepsis, Septic shock, Lactate kinetics, Mitochondrial respiration, Resuscitation, Cytokines, Multiple organ failure

## Abstract

**Background:**

A decrease in blood lactate levels (Lac) >10% during the first hours of resuscitation in sepsis is associated with better outcomes, but the mechanisms are unclear. Our objective was to investigate the relationship between the time course of Lac, inflammatory response, and mitochondrial respiration during experimental sepsis.

**Methods:**

Original data from two previously published studies were reanalyzed. In cohort 1, pigs were randomized to be resuscitated for 48 h starting at 6, 12, and 24 h, respectively, after fecal peritonitis induction (*n* = 8 each). Animals were categorized according to the decrease in Lac during the first 6 h of resuscitation (early if ≥10% [Lac ≥10%] or late if <10% or increased [Lac <10%]), and systemic hemodynamics, inflammatory parameters, and mitochondrial function were compared between groups. In a second group of animals with fecal peritonitis and 24 h of resuscitation (*n* = 16, cohort 2), abdominal regional Lac exchange was measured, and animals were categorized according to the decrease in Lac as in cohort 1.

**Results:**

Overall mortality was 20% (4 of 20) in the Lac ≥10% group and 60% (12 of 20) in the Lac <10% group (*p* = 0.022). In cohort 1, systemic hemodynamics were similar in the Lac ≥10% (*n* = 13) and Lac <10% (*n* = 11) groups. Plasma interleukin-6 levels increased during unresuscitated sepsis and decreased during resusciation in both groups, but they were lower at study end in the Lac ≥10% group (*p* = 0.047). Complexes I and II maximal (state 3) and resting (state 4) isolated brain mitochondrial respiration at study end was higher in the Lac ≥10% group than in the Lac <10% group, whereas hepatic, myocardial, and skeletal muscle mitochondrial respiration was similar in both groups. In cohort 2, mesenteric, total hepatic, and renal blood flow at study end was higher in the Lac ≥10% group (*n* = 7) than in the Lac <10% group (*n* = 9), despite similar cardiac output. Hepatic lactate influx and uptake in the Lac ≥10% group were approximately 1.5 and 3 times higher, respectively, than in the Lac <10% group (*p* = 0.066 for both).

**Conclusions:**

A decrease in Lac >10% during early resuscitation (6 h) after abdominal sepsis is associated with lower levels of plasma interleukin-6 and improved brain but not hepatic or muscle mitochondrial respiration. Blood flow redistribution to abdominal organs in animals with early decrease in Lac concentrations increases the potential to both deliver and extract Lac.

**Electronic supplementary material:**

The online version of this article (doi:10.1186/s13054-017-1691-4) contains supplementary material, which is available to authorized users.

## Background

Hyperlactatemia and lactic acidosis are widely accepted as strong predictors of multiple-organ dysfunction and death in different populations of critically ill patients [1–5]. Measurement of blood lactate (Lac) for the management of severe sepsis and septic shock has been strongly recommended since the first publication of the international sepsis guidelines [6, 7].

Although there are no single or direct interventions available to reverse hyperlactatemia, it has been proposed that Lac could be used as a guide during early resuscitation of patients with sepsis [8, 9]. It has been shown that a decrease in blood Lac levels >10% during the first 6 h of resuscitation is associated with reduced in-hospital mortality [10]. Interestingly, in another study, mortality was improved in the group with the goal of early Lac decrease despite the fact that achieved Lac values did not differ between the intervention and control groups [8].

In the last 2 decades, the assumption that high Lac levels represent a consequence of anaerobic metabolism has been challenged [11]. Other factors not related to insufficient tissue oxygen delivery may contribute to hyperlactatemia, such as impaired aerobic respiration, accelerated aerobic glycolysis, and reduced Lac uptake by the liver [12].

We recently demonstrated that treatment delay in fecal peritonitis increased inflammatory markers and Lac concentrations and was associated with impaired cerebral mitochondrial respiration at the end of the resuscitation period [13]. However, all these variables varied widely in individual animals [13]. In the present investigation, we hypothesized that animals with a greater decrease in blood Lac levels during the early resuscitation period would exhibit a less intense inflammatory response and preserve better mitochondrial respiration. To evaluate a possible contribution of gut and hepatic Lac handling to time course of blood Lac levels, we reanalyzed a second group of animals with fecal peritonitis where these measurements were available [14].

## Methods

We used original data from two previously published studies [13, 14]. The studies were performed in accordance with the National Institutes of Health Guide for the Care and Use of Experimental Animals and with the approval of the Animal Care Committee of the Canton of Bern, Switzerland.

The first study (cohort 1) was designed to address the impact of three different treatment delays (i.e., resuscitation initiated 6, 12, or 24 h after induction of fecal peritonitis) on disease severity, development of sepsis-associated organ dysfunction, and mitochondrial function [13]. In the second study (cohort 2), the impact of two different fluid resuscitation strategies (moderate-volume and high-volume expansion) on mortality, sepsis-associated organ dysfunction, and mitochondrial function in animals subjected to either fecal peritonitis or endotoxin infusion were evaluated [14]. For cohort 2, only data from animals challenged with fecal peritonitis were included. The full study protocols of the original studies can be found elsewhere [13, 14].

### Cohort 1

#### Experimental protocol

Fecal peritonitis was induced by peritoneal instillation of 2 g/kg body weight of autologous feces dissolved in 200 ml of 5% glucose solution. Thirty minutes after surgery, baseline (BL) measurements were taken. Then animals (*n* = 8 per group) were randomized to a sham control group or one of three groups in which resuscitation was started 6, 12, or 24 h after peritonitis induction. The 48-h resuscitation period consisted of broad-spectrum antibiotics (piperacillin/tazobactam [Tazobac®; Savoy, Manimajra, India] 2.25 g intravenously) administered every 8 h, volume expansion (alternating bolus of 150 ml of Ringer’s lactate and 6% hydroxyethyl starch 130/0.4 [HES]), vasopressors, and inotropes wherever necessary to reach predefined hemodynamic goals [13].

#### Blood gas analyses

Blood samples were drawn from an indwelling carotid artery catheter for Lac measurement (in millimoles per liter) (GEM Premier 3000 analyzer; Instrumentation Laboratory, Bedford, MA, USA) at BL and every 6 h during the resuscitation period. Inflammatory markers (plasma interleukin-6 [IL-6], tumor necrosis factor-α [TNF-α], and C-reactive protein [CRP]) were measured in blood samples drawn from the carotid artery at BL, immediately before starting resuscitation maneuvers (BR), and at the end of the experiment as previously described [13].

#### Mitochondrial function and skeletal muscle adenosine triphosphate content

At BL, BR, and the end of the experiment, tissue samples were taken from the right quadriceps muscle to assess mitochondrial function and skeletal muscle adenosine triphosphate (ATP) content as previously reported [13]. Additional tissue samples were taken from the brain, liver (left lobe), and heart (left ventricle) at the end of the experiment [13]. In animals that died earlier, the final tissue samples were taken when the animals were still alive, were receiving the maximal norepinephrine dose (1000 μg/h), and when mean arterial blood pressure approached 30 mmHg [13].

Oxygen consumption by complexes I, II, and IV was measured using high-resolution respirometry (Oxygraph-2 k; Oroboros Instruments, Innsbruck, Austria) and was expressed as picomoles per second per milligram of mitochondrial protein. State 3 represents active respiration after addition of adenosine diphosphate (ADP), whereas state 4 represents respiration after depletion of ADP. The respiratory control ratio, an indicator of coupling between respiration and ATP phosphorylation, was calculated by dividing state 3 by state 4 respiration rates [13]. The time course of blood Lac levels was defined as change in blood Lac levels in percent during 6 h of resuscitation [15].

### Cohort 2

#### Surgical preparation

With animals in supine position, a midline laparotomy was performed, and the abdominal cavity was exposed [14]. Catheters for pressure monitoring and blood sampling were inserted into the carotid, hepatic, and pulmonary arteries and into the jugular, hepatic, portal, mesenteric, splenic, and renal veins [14]. A large-bore catheter for fluid administration was inserted into the femoral vein. Ultrasound Doppler flow probes (Transonic® Systems Inc., Ithaca, NY, USA) were placed around the carotid, superior mesenteric, splenic, hepatic, celiac trunk, and renal arteries and around the portal vein. A drainage catheter was inserted into the urinary bladder. Finally, two large-bore drains were inserted into both flanks of the animals [14]. The surgical procedure was followed by a 12-h period of hemodynamic stabilization.

#### Experimental protocol

Fecal peritonitis was induced by peritoneal instillation through a peritoneal drainage tube of 1 g/kg body weight of autologous feces dissolved in 200 ml of 5% glucose solution [14]. The intraperitoneal drains were maintained clamped during the following 6 h. Before fecal peritonitis induction, animals were randomized to either moderate-volume (10 ml/kg/h Ringer’s lactate) or high-volume (15 ml/kg/h Ringer’s lactate + 5 ml/kg/h HES) (*n* = 8 each) fluid resuscitation for 24 h or until death, if earlier. Resuscitation started immediately after fecal peritonitis induction. Wherever necessary to reach a pulmonary arterial occlusion pressure >5 mmHg or urinary output >0.5 ml/kg/h, an additional fluid bolus (50 ml of HES) was given as long as stroke volume increased ≥10%. Vasopressors and inotropes were not used [14].

#### Blood measurements

Hemoglobin and oxygen saturation were analyzed at BL (before fecal peritonitis induction) and every 6 h thereafter (OSM3; Radiometer, Copenhagen, Denmark). At the same time, blood gases were measured and values corrected for temperature using a blood gas analyzer (ABL520; Radiometer), and arterial and venous blood Lac levels were measured with a Lac analyzer (YSI 2300 STAT PLUS; YSI Life Sciences, Yellow Springs, OH, USA) [14].

#### Calculations

In contrast to cohort 1, where resuscitation was delayed for 6–24 h, resuscitation in cohort 2 started immediately after fecal peritonitis was induced. Therefore, BL for Lac time-course analysis in cohort 2 was considered after 6 h of resuscitation, corresponding to the shortest resuscitation delay in cohort 1 and the time point of maximum Lac levels.

Systemic oxygen delivery and consumption were calculated according to standard formulas [16, 17]. Hepatic Lac influx (in micromoles per kilogram per minute) was calculated as follows: (portal venous Lac × portal vein blood flow) + (arterial Lac × hepatic arterial blood flow). Hepatic Lac efflux (in micromoles per kilogram per minute) was calculated as follows: hepatic venous Lac × (portal venous + hepatic arterial blood flow). Hepatic Lac uptake (in micromoles per kilogram per minute) was calculated as follows: hepatic Lac influx − hepatic Lac efflux. Other regional Lac exchanges (spleen, gut, kidney, and lung) were calculated as follows: regional Lac influx − regional Lac efflux. Whole-body venous efflux (in micromoles per kilogram per minute) was calculated as follows: cardiac output × mixed venous Lac concentration. Extrahepatic organ Lac efflux was calculated as follows: (cardiac output × mixed venous Lac concentration) − hepatic Lac efflux [17].

### Statistical analysis

In each cohort, animals were classified according to their time course of blood Lac levels: early (≥10% Lac decrease, Lac ≥10%) vs. late (<10% Lac decrease or increase, Lac <10%). All data are presented as mean ± SD, or as median with IQR in cases of non-normal distribution (tested by the Kolmogorov-Smirnov test).

Proportions between groups were compared with Fisher’s exact test. Survival analysis was performed using the Kaplan-Meier method, and the log-rank test was used for between-group comparisons. Continuous variables (administered treatments; fluid output and balance; brain, liver, and heart mitochondrial respiration) were compared between groups with an independent-samples *t* test or with the Mann-Whitney *U* test in cases of non-normal distribution.

Repeated measurements were assessed by analysis of variance using group as a between-subjects factor and time as a within-subjects factor. If a time × group interaction was detected, independent-samples *t* tests were performed at the end of the 6-h resuscitation period used for Lac kinetics and at the end of the experiment. In cases of non-normal distribution, time effects within each group were separately assessed by Friedman’s test followed by a Mann-Whitney *U* test at the end of the 6-h resuscitation period and at the end of the experiment for differences between groups. To account for testing twice, the significance level was reduced from 0.05 to 0.025 (Bonferroni correction). IBM SPSS Statistics version 21.0 software (IBM, Armonk, NY, USA) was used for statistical analyses, and Prism version 6.07 software (GraphPad Software, La Jolla, CA, USA) was used for graph plotting.

## Results

### Cohort 1

**Fig. 1:**
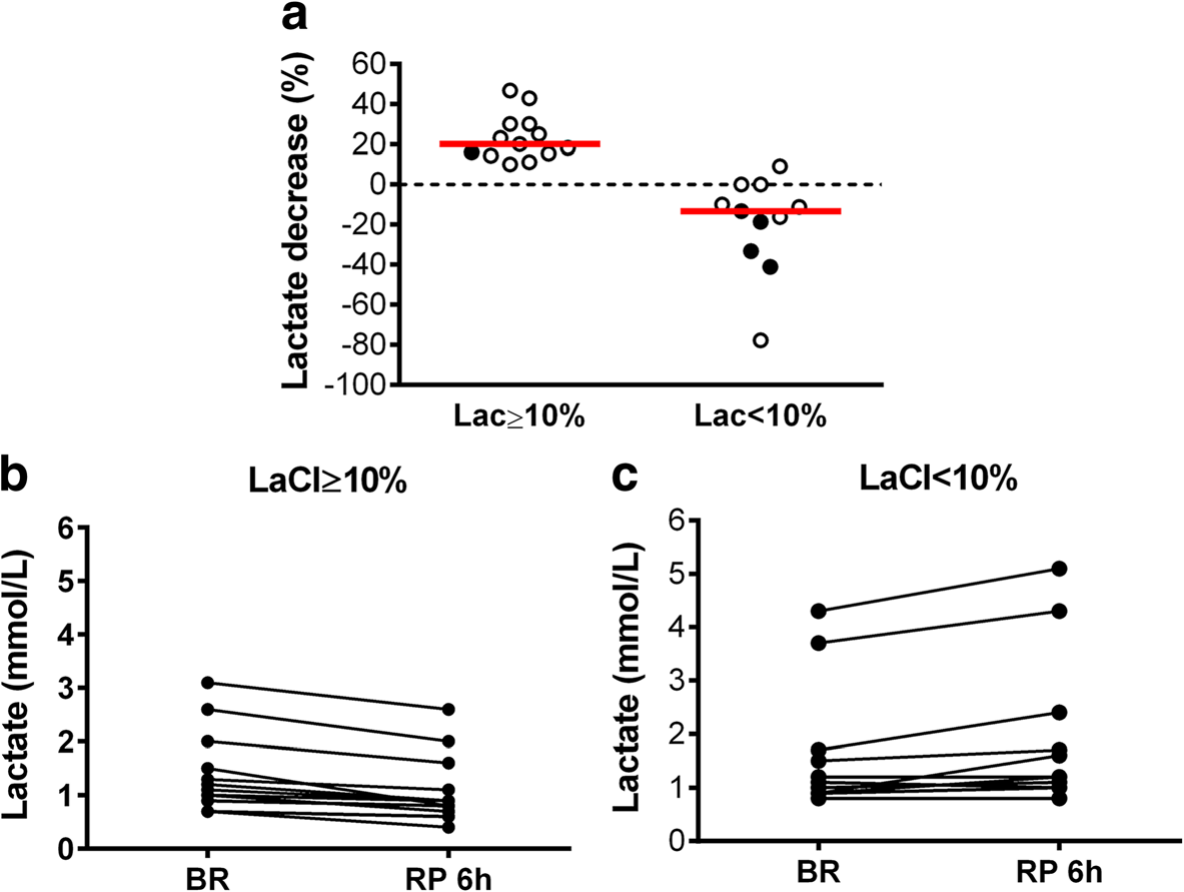
Time course of blood lactate (Lac) levels in cohort 1 animals. **a** Changes in blood Lac levels during the first 6 h of resuscitation accordingly to study group. **b** and **c** Individual arterial Lac levels before starting resuscitation maneuvers (BR) and after the first 6-h resuscitation period (RP 6 h) in the Lac ≥10% and Lac <10% groups. *Filled circles* in (**a**) represent animals that died during the 48-h resuscitation period. *Red horizontal bars* represent median values

Twenty-four domestic pigs of both sexes (mean ± SD weight 40.2 ± 3.8 kg) were analyzed. Of those, 13 of 24 (54.2%) had a decrease in blood Lac levels ≥10% (early or Lac ≥10% group) and 11 of 24 (45.8%) had a decrease in blood Lac levels <10% (late or Lac <10%) (Fig. 1). An additional.pdf file shows this in more detail (*see* Additional file 1: Table S1).

#### Mortality

**Fig. 2:**
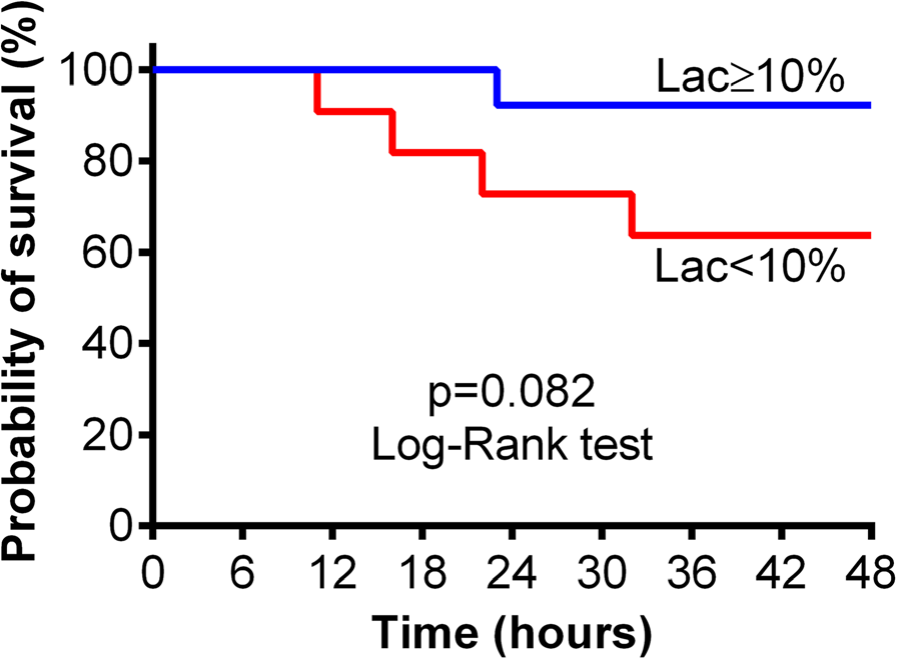
Kaplan-Meier curves for 48-h survival after the beginning of resuscitation in cohort 1

One of 13 animals (7.7%) in the Lac ≥10% group and 4 of 11 animals (36.4%) in the Lac <10% group died during the resuscitation period (*p* = 0.082) (Fig. 2).

#### Administered treatments

Doses of administered propofol, midazolam, fentanyl, resuscitation fluids, norepinephrine, and dobutamine, as well as net fluid balance, did not differ between the Lac ≥10% and Lac <10% groups (Table 1).

**Table 1:** Administered treatment, fluid output, and balance in cohort 1 study

Parameters	Lac ≥10%	Lac <10%	*p* value
Propofol, mg/kg/h	5.7 (5.1–6.2)	5.5 (4.9–5.9)	0.459^a^
Fentanyl^b^, μg/kg/h	4.9 (4.5–5.7)	5.2 (5.0–8.1)	0.072^a^
Midazolam, mg/kg/h	0.05 (0.04–0.06)	0.06 (0.05–00.7)	0.331^a^
Fluid bolus^c,d^, ml/kg/h	0.80 (0.53–1.24)	1.68 (0.50–2.82)	0.186^a^
Ringer’s lactate	0.45 (0.30–0.65)	0.84 (0.28–1.75)	0.186^a^
HES	0.39 (0.23–0.58	0.84 (0.21–1.07)	0.150^a^
Norepinephrine			
*n*/Total *n*, %	7/13 (53.8)	8/11 (72.7)	0.423^e^
Dose, μg/kg/minute	0.02 (0.01–0.07)	0.10 (0.06–0.22)	0.054^a^
Dobutamine			
*n*/Total *n*, %	2/13 (15.4)	5/11 (45.5)	0.182^e^
Dose, μg/kg/minute	1.89 (0.65–3.13)	0.44 (0.33–0.58)	0.190^a^
Total volume received^f^, ml/kg/h	3.61 (3.49–4.11)	4.12 (3.43–5.39)	0.303^a^
Urine output, ml/kg/h	1.00 (0.96–1.15)	1.21 (0.99–1.36)	0.134^a^
Gastric tube output, ml/kg/h	0.22 (0.05–0.30)	0.42 (0.22–0.71)	0.036^a^
Final balance^g^, ml/kg/h	2.48 (2.15–2.75)	2.45 (1.93–4.13)	1.000^a^

*HES* 6% Hydroxyethyl starch 130/0.4, *Lac* Lactate

Values are median (IQR) or *n*/total *n* (%) when indicated

^a^Mann-Whitney *U* test

^b^Sum of basic fentanyl infusion with additional bolus

^c^Sum of Ringer’s lactate and HES

^d^Administered during resuscitation period

^e^Fisher’s exact test

^f^Sum of Ringer’s lactate basal infusion + 50% glucose basal infusion + Ringer’s lactate bolus + HES

^g^Final balance = total volume received − (urine output + gastric tube output)

#### Hemodynamics and respiratory and acid-base parameters

Systemic and regional hemodynamics and oxygenation did not differ between the Lac ≥10% and Lac <10% groups (Table 2). Respiratory parameters (*see* Additional file 1: Table S2), as well as arterial blood gas analysis and hemoglobin levels (*see* Additional file 1: Table S3), were similar in both groups.

**Table 2:** Hemodynamics and arterial lactate levels in cohort 1

Parameters	Group	BL	BR	RP 6 h	End	*p* Value
Heart rate, beats/minute	Lac ≥10%	94 ± 20	160 ± 40	165 ± 26	132 ± 34	0.369^a^
	Lac <10%	97 ± 17	166 ± 30	161 ± 34	154 ± 33	
MAP, mmHg	Lac ≥10%	85 ± 12	75 ± 11	82 ± 13	65 ± 10	0.113^a^
	Lac <10%	88 ± 13	77 ± 19	71 ± 12	56 ± 20	
MPAP, mmHg	Lac ≥10%	14 ± 3	17 ± 3	17 ± 3	21 ± 5	0.594^a^
	Lac <10%	15 ± 6	17 ± 5	20 ± 6	22 ± 4	
CVP, mmHg	Lac ≥10%	4 ± 1	3 ± 2	3 ± 2	7 ± 2	0.632^a^
	Lac <10%	5 ± 3	2 ± 2	3 ± 2	8 ± 4	
Cardiac output, ml/kg/minute	Lac ≥10%	131 ± 14	106 ± 16	120 ± 20	151 ± 32	0.618^a^
	Lac <10%	132 ± 22	119 ± 33	122 ± 24	144 ± 46	
SVRI, mmHg, L/kg/minute	Lac ≥10%	629 ± 112	696 ± 146	677 ± 154	393 ± 92	0.322^a^
	Lac <10%	654 ± 171	652 ± 164	575 ± 164	326 ± 73	
SvO_2_, %	Lac ≥10%	55 ± 8	59 ± 11	64 ± 9	63 ± 12	0.264^a^
	Lac <10%	53 ± 9	57 ± 6	60 ± 6	52 ± 18	
DO_2_, ml/minute	Lac ≥10%	625 ± 99	684 ± 116	739 ± 76	653 ± 151	0.796^a^
	Lac <10%	610 ± 148	702 ± 133	697 ± 156	609 ± 197	
VO_2_, ml/minute	Lac ≥10%	273 ± 63	270 ± 86	249 ± 79	220 ± 81	0.987^a^
	Lac <10%	275 ± 61	281 ± 48	256 ± 60	234 ± 59	
O_2_ER	Lac ≥10%	0.44 ± 0.08	0.40 ± 0.11	0.34 ± 0.10	0.34 ± 0.13	0.511^a^
	Lac <10%	0.46 ± 0.09	0.41 ± 0.05	0.37 ± 0.06	0.43 ± 0.18	
Fractional carotid artery blood flow, %	Lac ≥10%	5.8 ± 1.9	4.7 ± 1.8	4.6 ± 1.5	4.4 ± 1.5	0.340^a^
	Lac <10%	5.8 ± 1.2	5.4 ± 1.3	5.6 ± 1.3	5.0 ± 2.0	
Fractional femoral artery blood flow, %	Lac ≥10%	3.3 ± 1.1	2.0 ± 0.5	1.7 ± 0.5	1.9 ± 0.5	0.499^a^
	Lac <10%	3.3 ± 0.8	2.1 ± 0.7	2.2 ± 0.9	2.1 ± 0.8	
Arterial Lac, mmol/L	Lac ≥10%	0.8 ± 0.1	1.4 ± 0.7	1.1 ± 0.6	1.1 ± 0.7	0.124^a^
	Lac <10%	0.9 ± 0.3	1.6 ± 1.2	1.9 ± 1.4	2.3 ± 2.5	

*Abbreviations: BL* Baseline, *BR* Immediately before start resuscitation, *RP* Resuscitation period, *End* End of the experiment (at 48 h of resuscitation or before death if earlier), *MAP* Mean arterial blood pressure, *MPAP* Mean pulmonary arterial pressure, *CVP* Central venous pressure, *SVRI* Systemic vascular resistance index, *SvO*_*2*_ Mixed venous oxygen saturation, *DO*_*2*_ Oxygen delivery, *VO*_*2*_ Oxygen consumption, *O*_*2*_ *ER* Oxygen extraction ratio

Values represent mean ± SD

^a^Time × group interaction with repeated-measures analysis of variance including all time points

#### Inflammatory markers and skeletal muscle ATP

Plasma IL-6 levels increased during untreated sepsis and decreased during the resuscitation period in both groups, but they were lower at the end of the study in the Lac ≥10% group than in the Lac <10% group (*p* = 0.047) (Table 3). Plasma TNF-α levels, CRP, and skeletal muscle ATP content did not differ between groups (Table 3).

**Table 3:** Inflammatory markers and skeletal muscle adenosine triphosphate content in cohort 1

Parameters	Group	BL	BR	End	*p* Value
IL-6, pg/ml	Lac ≥10%	0 (0–0)	854 (481–990)	45 (37–204)	0.003^a^
	Lac <10%	16 (0–180)	873 (531–1983)	166 (128–310)	<0.001^a^
					0.047^b^
CRP, pg/ml	Lac ≥10%	1.6 (0.6–2.9)	10.7 (5.6–12.8)	19.2 (9.9–39.8)	0.139^c^
	Lac <10%	3.2 (1.8–5.7)	16.7 (4.9–31.1)	16.7 (13.5–33.3)	
TNF-α, pg/ml	Lac ≥10%	53 (38–55)	119 (94–169)	72 (47–88)	0.579^c^
	Lac <10%	47 (37–67)	178 (120–219)	92 (57–144)	
ATP content, μmol/g wet tissue weight	Lac ≥10%	2.1 (1.2–2.3)	2.3 (1.8–3.1)	0.6 (0.4–0.9)	0.081^c^
	Lac <10%	2.8 (1.8–4.5)	3.3 (1.5–4.8)	0.6 (0.3–0.7)	

*Abbreviations: ATP* Adenosine triphosphate, *BL* Baseline, *BR* Immediately before start resuscitation, *End* End of the experiment (at 48 h of resuscitation or before death if earlier), *IL-6* Interleukin-6, *CRP* C-reactive protein, *TNF-α* Tumor necrosis factor-α

Values represent median (IQR)

^a^Time effect with Friedman’s test including all time points

^b^Mann-Whitney *U* test at end of study

^c^Time × group interaction with repeated-measures analysis of variance including all time points

#### Mitochondrial respiration

**Fig. 3:**
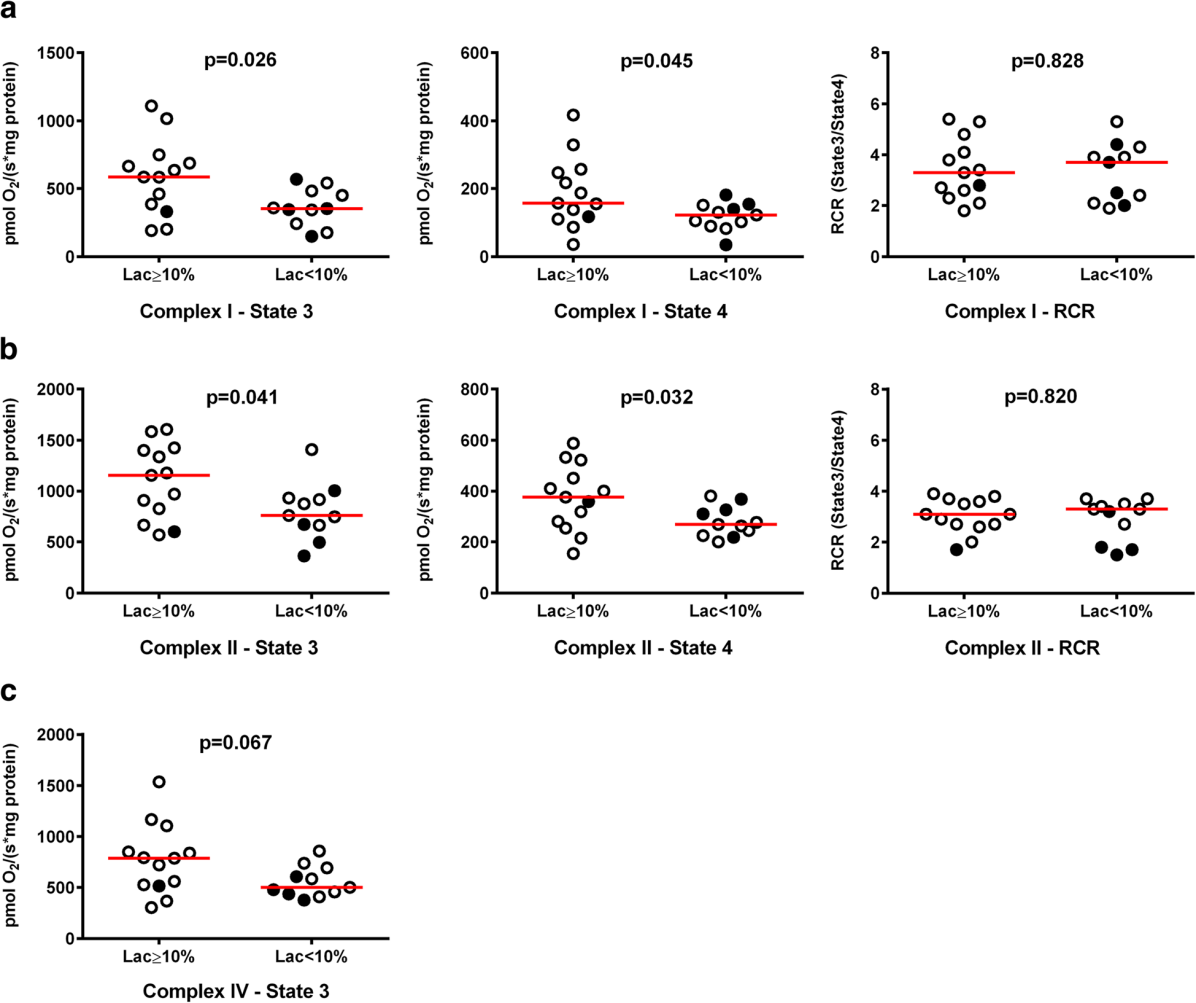
Isolated brain mitochondrial respiration in cohort 1 animals. *Red horizontal bars* represent median values. *Black filled circles* represent animals that died during the resuscitation period. *p* Values provided were calculated with an independent-samples *t* test or the Mann-Whitney *U* test. State 3 represents active respiration after addition of adenosine diphosphate (ADP), and state 4 represents the respiration after depletion of ADP. The respiratory control ratio (RCR) was calculated by dividing state 3 by state 4 respiration rates. **a** Complex I. **b** Complex II. **c** Complex – IV

Complexes I and II maximal (state 3) and resting (state 4) isolated brain mitochondrial respiration at the end of the experiment were higher in the Lac ≥10% group than in the Lac <10% group (Fig. 3 and Additional file 1: Table S4). Isolated skeletal muscle (*see* Additional file 1: Table S5), liver (*see* Additional file 1: Table S6), and heart (*see* Additional file 1: Table S7) mitochondrial respiration did not differ between the groups.

### Cohort 2

Sixteen domestic pigs of both sexes (mean ± SD weight 41.6 ± 2.7 kg) were included in the cohort 2 analysis. Of those, 7 of 16 (43.8%) exhibited a decrease in blood Lac levels ≥10% (early group), and 9 of 16 (56.2%) had a decrease in blood Lac levels <10% (late group) (*see* Additional file 1: Table S8 and Figure S1).

#### Mortality

Three of seven animals (42.9%) in the early (Lac ≥10%) group and eight of nine animals (88.9%) in the late (Lac <10%) group died during the resuscitation period (*p* = 0.006) (Additional file 1: Figure S2).

#### Hemodynamics

Systemic hemodynamics did not differ between the groups during the study period (*see* Additional file 1: Table S9).

#### Regional blood flow and Lac exchange

Superior mesenteric artery blood flow increased and renal artery blood flow decreased in both groups during the 24 h of resuscitation, but both flows were higher at the end in the Lac ≥10% group than in the Lac <10% group (Table 4). Similarly, total hepatic blood flow was higher in the Lac ≥10% group than in the Lac <10% group at the study end (Table 4). Fractional regional blood flows are presented in Additional file 1: Table S10.

**Table 4:** Regional blood flow in cohort 2

Parameters	Group	BL	3 h	6 h	12 h	End	*p* value	Time × group *p* value^a^	Time *p* value^a^	Group *p* value^a^
Carotid artery	Lac ≥10%	4.2 ± 0.9	3.8 ± 1.1	3.6 ± 0.5	3.2 ± 0.7	5.7 ± 2.6	0.030^b^	0.077	0.458	0.078
	Lac <10%	3.9 ± 0.9	4.1 ± 1.3	4.1 ± 1.4	5.0 ± 2.0	4.9 ± 1.6	0.457^c^			
							0.028^c^			
Superior mesenteric artery	Lac ≥10%	18.4 ± 8.0	17.7 ± 9.8	17.5 ± 6.6	18.8 ± 9.6	26.6 ± 11.0	0.010^b^	0.858	0.179	0.807
	Lac <10%	13.7 ± 2.9	15.7 ± 3.1	16.6 ± 3.4	18.2 ± 4.1	15.3 ± 7.6	0.028^c^			
Celiac trunk	Lac ≥10%	9.2 ± 4.6	10.8 ± 3.4	9.8 ± 4.4	12.5 ± 3.9	11.8 ± 5.9	0.191^b^	0.067	0.221	0.067
	Lac <10%	8.8 ± 3.7	9.2 ± 4.1	9.9 ± 4.2	9.3 ± 5.0	7.0 ± 2.1				
Hepatic artery	Lac ≥10%	4.2 ± 3.7	5.2 ± 2.9	5.0 ± 3.0	6.3 ± 3.6	5.5 ± 2.6	0.232^b^	0.100	0.149	0.142
	Lac <10%	3.7 ± 1.8	3.0 ± 1.6	3.7 ± 1.8	3.6 ± 2.3	2.5 ± 0.9				
Portal vein	Lac ≥10%	20.5 ± 7.0	20.5 ± 11.5	19.2 ± 6.7	20.5 ± 8.4	27.4 ± 11.6	0.006^b^	0.717	0.408	0.488
	Lac <10%	19.3 ± 5.4	22.3 ± 5.6	21.8 ± 4.3	22.3 ± 6.0	17.3 ± 10.0	0.083^c^			
Total hepatic^d^	Lac ≥10%	24.8 ± 8.2	25.7 ± 12.1	24.2 ± 8.0	26.8 ± 9.4	32.8 ± 13.0	0.006^b^	0.371	0.224	0.965
	Lac <10%	23.0 ± 5.4	25.3 ± 6.1	25.4 ± 4.7	25.9 ± 7.8	19.8 ± 10.1	0.040^c^			
Spleen artery	Lac ≥10%	1.1 ± 0.4	1.1 ± 1.1	1.0 ± 0.8	1.2 ± 1.0	1.2 ± 1.2	0.049^b^	0.115	0.341	0.766
	Lac <10%	1.1 ± 0.3	1.5 ± 1.2	1.3 ± 0.8	1.2 ± 1.0	0.5 ± 0.2	0.167^c^			
Renal artery	Lac ≥10%	5.2 ± 1.9	5.1 ± 1.4	4.4 ± 1.0	5.0 ± 1.0	3.9 ± 1.3	0.036^b^	0.059	0.409	0.782
	Lac <10%	5.8 ± 2.0	5.4 ± 2.1	5.7 ± 2.9	4.3 ± 2.7	2.1 ± 1.4	0.018^c^			

*BL* baseline, *End* End of experiment after 24 h of randomization or before death, if earlier

Values represent mean ± SD. All flows are in milliliters per kilogram per minute

^a^ *p* Values were calculated with repeated-measures analysis of variance including time points 6 h and 12 h for time × group interaction, time effect, and group effect

^b^Time × group interaction with repeated-measures analysis of variance including all time points

^c^Independent-samples *t* test at end of study

^d^Sum of hepatic artery and portal vein blood flows

Hepatic Lac delivery (hepatic Lac influx) and uptake in the Lac ≥10% group were approximately 1.5 and 3 times higher, respectively, than in the Lac <10% group at the end of the study, although statistical significance was not reached (*p* = 0.066 for both) (Table 5). Gut, spleen, kidney, and lung Lac uptake did not differ between groups (Table 5).

**Table 5:** Lac influx, efflux, and uptake in cohort 2

Parameters	Group	BL	3 h	6 h	12 h	End	*p* value	Time × group *p* value^a^	Time *p* value^a^	Group *p* value^a^
Hepatic Lac uptake	Lac ≥10%	10.3 (7.0 to 14.8)	7.8 (5.5 to 15.7)	10.6 (2.3 to 11.4)	15.4 (8.0 to 17.3)	23.0 (8.9 to 26.3)	0.066^b^	0.389	0.221	0.754
	Lac <10%	12.1 (11.9 to 19.5)	15.0 (10.2 to 22.5)	11.4 (9.3 to 12.7)	7.0 (3.5 to 22.3)	7.5 (6.6 to 11.2)				
Hepatic Lac influx	Lac ≥10%	24.8 (19.0 to 30.2)	30.8 (18.8 to 57.8)	23.9 (19.0 to 49.4)	39.4 (13.6 to 47.1)	32.6 (29.0 to 59.6)	0.066^c^			
	Lac <10%	27.1 (19.3 to 45.0)	35.2 (16.5 to 56.5)	25.5 (18.3 to 33.7)	32.5 (17.5 to 45.2)	20.3 (10.1 to 32.5)	0.321^c^			
Hepatic Lac efflux	Lac ≥10%	12.7 (8.1 to 26.3)	15.0 (5.5 to 52.4)	21.3 (7.7 to 35.9)	26.0 (6.8 to 27.1)	22.1 (10.1 to 30.3)	0.645^b^	0.576	0.036	0.560
	Lac <10%	12.4 (9.1 to 23.7)	14.7 (4.7 to 38.8)	15.4 (8.1 to 20.9)	19.9 (14.0 to 32.3)	14.3 (3.5 to 21.6)				
Gut Lac uptake	Lac ≥10%	−2.6 (−2.9 to 0.3)	−2.2 (−2.8 to −2.1)	−1.7 (−3.9 to 0.3)	−2.4 (−6.8 to 0.5)	−3.8 (−9.9 to −0.6)	0.942^b^	0.440	0.779	0.299
	Lac <10%	−1.4 (−3.7 to 5.2)	−3.3 (−10.7 to 1.3)	−2.9 (−4.0 to −0.9)	−4.7 (−11.6 to −1.8)	−2.1 (−3.8 to −0.4)				
Spleen Lac uptake	Lac ≥10%	−0.2 (−0.7 to −0.2)	−0.2 (−0.6 to −0.1)	−0.2 (−1.1 to −0.1)	−0.1 (−0.9 to −0.1)	0.0 (−0.4 to 0.2)	0.185^b^	0.465	0.347	0.911
	Lac <10%	−0.1 (−0.3 to 0.1)	−0.1 (−0.3 to 0.1)	−0.3 (−1.6 to −0.1)	−0.1 (−0.7 to 0.0)	−0.1 (−0.2 to 0.0)				
Kidney Lac uptake	Lac ≥10%	−0.4 (−1.0 to 0.0)	−1.0 (−1.9 to −0.7)	−0.4 (−2.3 to 0.2)	−0.5 (−2.5 to 1.1)	0.3 (0.1 to 0.9)	0.110^b^	0.743	0.546	0.921
	Lac <10%	−0.1 (−0.8 to 2.5)	0.4 (−2.0 to 1.7)	−0.6 (−2.1 to 0.2)	−0.2 (−0.7 to 0.4)	−0.4 (−1.0 to 0.3)				
Lung Lac uptake	Lac ≥10%	−2.0 (−8.3 to 5.4)	−1.1 (−5.9 to 1.6)	−4.4 (−19.8 to 2.7)	−5.4 (−11.9 to 4.3)	0.0 (−5.4 to 108.3)	0.622^c^			
	Lac <10%	4.8 (−3.0 to 7.2)	−4.1 (−18.3 to 25.7)	−0.1 (−6.5 to 5.4)	−6.5 (−17.8 to 3.2)	−5.6 (−32.2 to 5.3)	0.525^c^			
Whole-body venous Lac efflux	Lac ≥10%	76.9 (61.6 to 116.3)	84.0 (57.1 to 174.6)	105.4 (93.8 to 162.8)	119.7 (47.6 to 123.6)	104.3 (79.7 to 131.9)	0.635^b^	0.144	0.253	0.692
	Lac <10%	98.3 (59.2 to 38.6)	107.3 (61.7 to 215.6)	90.2 (76.1 to 109.1)	104.3 (84.3 to 188.1)	110.5 (69.4 to 327.1)				
Extrahepatic organ Lac efflux	Lac ≥10%	69.6 (56.5 to 90.0)	59.1 (39.1 to 137.2)	97.0 (72.5 to 117.6)	91.4 (40.2 to 102.4)	82.0 (71.0 to 101.6)	0.346^b^	0.077	0.883	0.397
	Lac <10%	66.3 (50.0 to 123.5)	92.6 (53.3 to 176.8)	74.8 (61.5 to 94.8)	87.9 (66.8 to 154.6)	102.9 (63.9 to 191.1)				

*BL* baseline, *End* End of experiment after 24 h of randomization or before death, if earlier, *Lac* Lactate

Values represent median (IQR). All Lac uptake and efflux values are in micromoles per kilogram per minute

^a^ *p* Values were calculated with repeated-measures analysis of variance including time points 6 h and 12 h for time × group interaction, time effect, and group effect

^b^Time × group interaction was calculated with repeated-measures analysis of variance including all time points

^c^Time effect was calculated with Friedman’s test including all time points

#### Hepatic function and oxygen consumption

Serum alanine aminotransferase, aspartate aminotransferase, total bilirubin, and hepatic oxygen consumption did not differ between groups (*see* Additional file 1: Table S11).

## Discussion

The main finding of the present study was that a decrease in blood Lac levels ≥10% during the first 6 h of resuscitation, regardless of the delay in starting resuscitation, was associated with lower levels of IL-6 and higher brain mitochondrial respiration after 48 h of resuscitation. The enhanced decrease in Lac levels (early group) was associated with higher absolute and relative (fraction of cardiac output) abdominal blood flows favoring higher hepatic Lac delivery and uptake.

Lac and inflammation have been linked recently in different contexts and diseases, including in cancer [18], asthma [19], and abdominal surgery [20]. During experimentally induced systemic inflammatory conditions (e.g., acute pancreatitis and acute hepatitis) Lac may downregulate Toll-like receptor 4, NLRP3 (NACHT, LRR, and PYD domains-containing protein 3) inflammasome, and concomitantly IL-1β [21]. Nguyen et al. divided 220 patients with sepsis into 4 different groups according to Lac kinetics and showed an inverse correlation between IL-1 receptor antagonist, IL-6, IL-8, IL-10, TNF-α, intercellular adhesion molecule 1, high-mobility group box 1 protein, D-dimer, and caspase-3 concentrations and Lac clearance quartiles [22]. We do not have data to evaluate whether early decreases in blood Lac levels reduced the extent of inflammation or whether a less aggravated inflammatory response enhanced Lac handling. It is possible that pronounced inflammation directly or indirectly impedes the ability of mitochondria to use Lac as a crucial alternative source of energy [23, 24]. In a study of endotoxemic sheep, both esmolol and dexmedetomidine improved exogenous Lac clearance [23]. The study authors suggested that the anti-inflammatory properties of these two drugs may have contributed to this finding.

The importance of Lac as a mitochondrial substrate was reinforced with the Lac shuttle theory [24] and, very recently, with the demonstration that cytosolic Lac can be directly metabolized within mitochondria by an intrinsic nicotinamide adenine dinucleotide-dependent L-lactate dehydrogenase [25, 26]. Increased use of Lac by cerebral mitochondria in animals in our Lac ≥10% group could theoretically explain better mitochondrial respiration.

Whereas mitochondrial respiration was higher in brain biopsies of our Lac ≥10% group animals, it was not in biopsies of heart, liver, and skeletal muscle. On one hand, in sepsis, use of substrates and mitochondrial respiration efficiency vary between organs [27]. On the other hand, brain mitochondria may be especially vulnerable to toxic products released as a consequence of septic liver dysfunction [28]. For example, hyperammonemia can compromise energy metabolism by inhibition of α-ketoglutarate dehydrogenase in cerebral isolated mitochondria [29, 30].

In our Lac ≥10% group, state 3 as well as state 4 respiration was slightly higher than in our Lac <10% group, indicating mild uncoupling of respiration due to proton leak. Mild uncoupling is not associated with decreasing ATP synthesis rates [31]. Proton leak across the mitochondrial inner membrane may be mediated by components of the mitochondrial inner membrane proteins, such as uncoupling proteins and adenine nucleotide translocase [32]. Mild uncoupling can be helpful in protecting against oxidative damage by reducing reactive oxygen species (ROS) production [33, 34]. It has been shown that ROS can activate the transcription factor nuclear factor-κB (NF-κB) [35] and that activation of IL-6 gene expression occurs through NF-κB [36], which may explain higher levels of IL-6 in our Lac <10% group.

Numerically higher hepatic Lac uptake in the Lac ≥10% group of cohort 2 may indicate better-preserved liver function in this group. We cannot judge the effect of increased liver perfusion (and hence Lac delivery) on liver Lac uptake. In a short-term endotoxemic shock model in sheep, exogenous Lac clearance decreased despite maintained liver perfusion [37]. Nevertheless, the authors in that study did not address the relationship between (widely varying) liver perfusion and exogenous Lac clearance in their endotoxemic animals [37]. It has been shown that, in sepsis, the gut releases, among other substances, adrenomedullin, a potent vasodilatory peptide [38]. In our study, the other monitored organs did not play a major role in Lac uptake.

Our findings must be interpreted with caution, mainly regarding inadvertent attempts to establish cause-and-effect relationships, because this was a hypothesis-generating study. On one hand, the different design of the two analyzed studies is a limitation. On the other hand, mortality trend differences were consistent between the early (Lac ≥10%) and late (Lac <10%) groups when resuscitation followed immediately after the septic insult or was delayed. Thus, another limitation is that we did not measure Lac uptake of the brain and the heart, two organs where mitochondrial Lac use has been demonstrated previously [23, 24]. Furthermore, in vitro mitochondrial respiration may not directly reflect in vivo conditions in diseases where substrate availability may be altered. The association between Lac time course, inflammation, and brain mitochondrial function in a clinically relevant model of sepsis suggests that inflammation may modify brain mitochondrial respiration. To evaluate this further, changes in brain tissue inflammation and mitochondrial respiration should be studied.

## Conclusions

A decrease in blood Lac level ≥10% is associated with lower levels of plasma IL-6 and better brain mitochondrial respiration during the first 6 h of resuscitation after experimental sepsis induced by fecal peritonitis. Increased liver Lac uptake may account at least partially for the differences.

## Additional file

Additional file 1: Table S1. Composition of study groups in cohort 1. **Table S2.** Respiratory system variables in cohort 1. **Table S3.** Arterial blood gas analysis and arterial hemoglobin levels in cohort 1. **Table S4.** Isolated brain mitochondrial respiration in cohort 1. **Table S5.** Skeletal muscle-dependent mitochondrial respiration in cohort 1. **Table S6.** Isolated liver mitochondrial respiration in cohort 1. **Table S7.** Isolated heart mitochondrial respiration in cohort 1. **Table S8.** Composition of study groups in cohort 2. **Table S9.** Systemic hemodynamics and arterial lactate levels in cohort 2. **Table S10.** Fractional regional blood flow in cohort 2. **Table S11.** Liver tests and hepatic oxygen consumption in cohort 2. **Figure S1.** Time course of blood lactate levels in cohort 2. **Figure S2.** Kaplan-Meier curves for 24-h survival after randomization in cohort 2. (DOC 389 kb)

## Abbreviations

ADP: Adenosine diphosphate
ATP: Adenosine triphosphate
BL: Baseline
BR: Before starting resuscitation maneuvers
CRP: C-reactive protein
CVP: Central venous pressure
DO_2_: Oxygen delivery
END: End of the experiment
HES: 6% Hydroxyethyl starch 130/0.4
IL-6: Interleukin-6
Lac: Lactate
MAP: Mean arterial pressure
MPAP: Mean pulmonary arterial pressure
NF-κB: Nuclear factor-κB
O_2_ER: Systemic oxygen extraction ratio
RCR: Respiratory control ratio (state 3 respiration divided by state 4 respiration)
ROS: Reactive oxygen species
RP: Resuscitation period
SvO_2_: Mixed venous oxygen saturation
SVRI: Systemic vascular resistance index
TNF-α: Tumor necrosis factor α
VO_2_: Oxygen consumption
