# Supplementary material for: Time course of blood lactate levels, inflammation, and mitochondrial function in experimental sepsis
Source: Crit Care. 2017 May 12;21:105. doi: 10.1186/s13054-017-1691-4 (PMC5429522; doi:10.1186/s13054-017-1691-4)
Supplement: Additional file 1: Table S1. — Composition of study groups in cohort 1. Table S2. Respiratory system variables in cohort 1. Table S3. Arterial blood gas analysis and arterial hemoglobin levels in cohort 1. Table S4. Isolated brain mitochondrial respiration in cohort 1. Table S5. Skeletal muscle-dependent mitochondrial respiration in cohort 1. Table S6. Isolated liver mitochondrial respiration in cohort 1. Table S7. Isolated heart mitochondrial respiration in cohort 1. Table S8. Composition of study groups in cohort 2. Table S9. Systemic hemodynamics and arterial lactate levels in cohort 2. Table S10. Fractional regional blood flow in cohort 2. Table S11. Liver tests and hepatic oxygen consumption in cohort 2. Figure S1. Time course of blood lactate levels in cohort 2. Figure S2. Kaplan-Meier curves for 24-h survival after randomization in cohort 2. (DOC 389 kb) [file 13054_2017_1691_MOESM1_ESM.doc]

**TIME COURSE OF BLOOD LACTATE LEVELS, INFLAMMATION AND MITOCHONDRIAL FUNCTION IN EXPERIMENTAL SEPSIS**

**Additional file 1**

**Authors:** Thiago Domingos Corrêa*, Adriano José Pereira*, Sebastian Brandt, Madhusudanarao Vuda, Siamak Djafarzadeh, Jukka Takala and Stephan Mathias Jakob

* Contributed equally

**Table S1.** Composition of study groups in cohort-1.

| Study groups* | Lac≥10% | Lac<10% | p value |
| --- | --- | --- | --- |
| n/total n (%) | 13/24 (54.2) | 11/24 (45.8) | 0.459 |
| 6hs | 6 (46.2) | 2 (18.2) |  |
| 12hs | 3 (23.1) | 5 (45.5) |  |
| 24hs | 4 (30.8) | 4 (36.3) |  |

Values represent n (%).* Time elapsed between peritonites induction and the beginning of resuscitation. P value with Fisher’s exact test.

**Table S2.** Respiratory system variables in cohort-1.

| **Parameters** | **Group** | **BL** | **BR** | **RP 6h** | **End** | **p value** |
| --- | --- | --- | --- | --- | --- | --- |
| Respiratory rate (ipm) | Lac≥10% | 21± 1 | 24 ± 3 | 24 ± 4 | 28 ± 6 | 0.800^a^ |
|  | Lac<10% | 22 ± 5 | 24 ± 4 | 25 ± 5 | 29 ± 5 |  |
| Tidal volume (ml/kg) | Lac≥10% | 8.4 ± 0.5 | 8.4 ± 0.6 | 8.5 ± 0.6 | 8.5 ± 0.8 | 0.984^a^ |
|  | Lac<10% | 8.1 ± 0.4 | 8.1 ± 0.5 | 8.2 ± 0.7 | 8.2 ± 0.9 |  |
| PEEP (cm H_2_0) | Lac≥10% | 5 ± 0 | 5 ± 0 | 5 ± 0 | 9 ± 4 | <0.001^b^ |
|  | Lac<10% | 5 ± 0 | 5 ± 0 | 6 ± 2 | 9 ± 3 | <0.001^c^ |
|  |  |  |  |  |  | 0.277**^d^** |
|  |  |  |  |  |  | 0.691**^e^** |
| PaO_2_/FiO_2_ | Lac≥10% | 456 ± 41 | 420 ± 53 | 394 ± 80 | 240 ± 79 | 0.075^a^ |
|  | Lac<10% | 476 ± 69 | 388 ± 42 | 326 ± 102 | 186 ± 63 |  |

Values represent mean ± SD. BL = baseline, BR = immediately before start resuscitation, RP = resuscitation period, End = end of the experiment (at 48 hours of resuscitation or before death if earlier), PEEP = positive end-expiratory pressure and PaO_2_/FiO_2_ = arterial oxygen partial pressure and fraction of inspired oxygen relationship, a = time-group interaction with repeated measures analysis of variance including all time points, b and c = time effect with Friedman’s test including all time points, d = Mann-Whitney U-test at RP 6h and e = Mann-Whitney U-test at END.

**Table S3.** Arterial blood gas analysis and arterial hemoglobin levels in cohort-1.

| **Parameters** | **Group** | **BL** | **BR** | **RP 6h** | **End** | **p value** |
| --- | --- | --- | --- | --- | --- | --- |
| pH | Lac≥10% | 7.52 ± 0.03 | 7.45 ± 0.06 | 7.47 ± 0.03 | 7.47 ± 0.07 | 0.046^a^ |
|  | Lac<10% | 7.54 ± 0.03 | 7.42 ± 0.06 | 7.41 ± 0.09 | 7.38 ± 0.15 | 0.079^b^ |
|  |  |  |  |  |  | 0.103^c^ |
| Bicarbonate (mmol/L) | Lac≥10% | 32.5 ± 1.6 | 29.1 ± 2.8 | 29.7 ± 2.3 | 29.8 ± 3.3 | 0.100^a^ |
|  | Lac<10% | 33.0 ± 1.2 | 26.7 ± 3.0 | 27.0 ± 2.8 | 28.5 ± 4.8 |  |
| Base excess (mmol/L) | Lac≥10% | 9.5 ± 1.9 | 5.8 ± 3.4 | 6.7 ± 2.3 | 6.3 ± 3.9 | 0.075^a^ |
|  | Lac<10% | 10.3 ± 1.0 | 3.3 ± 3.6 | 3.3 ± 4.1 | 3.7 ± 6.5 |  |
| PaCO_2_ (mm Hg) | Lac≥10% | 40.2 ± 2.8 | 43.2 ± 3.5 | 41.7 ± 3.9 | 42.2 ± 4.2 | 0.014^a^ |
|  | Lac<10% | 39.3 ± 3.6 | 42.5 ± 4.2 | 43.6 ± 6.3 | 49.6 ± 10.6 | 0.367^b^ |
|  |  |  |  |  |  | 0.050^c^ |
| PaO_2_ (mm Hg) | Lac≥10% | 137 ± 12 | 129 ± 16 | 126 ± 21 | 112 ± 16 | 0.106^a^ |
|  | Lac<10% | 143 ± 21 | 129 ± 21 | 115 ± 22 | 97 ± 17 |  |
| Arterial hemoglobin (g/dl) | Lac≥10% | 8.8 ± 0.7 | 12.2 ± 1.8 | 11.8 ± 0.9 | 8.3 ± 0.9 | 0.205^a^ |
|  | Lac<10% | 8.9 ± 1.0 | 12.0 ± 1.8 | 11.4 ± 1.3 | 9.1 ± 1.6 |  |

Values represent mean ± SD. BL = baseline, BR = immediately before start resuscitation, RP = resuscitation period, End = end of the experiment (at 48 hours of resuscitation or before death if earlier), PaO_2_ = arterial oxygen partial pressure, PaCO_2_ = arterial carbon dioxide partial pressure, a = time-group interaction with repeated measures analysis of variance including all time points, b = independent samples t-test at RP 6h and c = independent samples t-test End.

**Table S4:** Isolated brain mitochondrial respiration in cohort-1.

| **Parameters** | **Group** | **Median (IQR)** | **P value** |
| --- | --- | --- | --- |
| **Complex 1** |  |  |  |
| State 3 | Lac≥10% | 586 (386-688) | 0.026**^a^** |
|  | Lac<10% | 353 (242-483) |  |
| State 4 | Lac≥10% | 157 (117-247) | 0.045**^a^** |
|  | Lac<10% | 122 (89-151) |  |
| RCR | Lac≥10% | 3.3 (2.6-4.1) | 0.828**^a^** |
|  | Lac<10% | 3.7 (2.1-4.3) |  |
| **Complex 2** |  |  |  |
| State 3 | Lac≥10% | 1156 (828-1401) | 0.041**^a^** |
|  | Lac<10% | 761 (664-932) |  |
| State 4 | Lac≥10% | 376 (281-451) | 0.032**^a^** |
|  | Lac<10% | 269 (225-326) |  |
| RCR | Lac≥10% | 3.1 (2.7-3.6) | 0.820**^b^** |
|  | Lac<10% | 3.3 (1.8-3.5) |  |
| **Complex 4** |  |  |  |
| State 3 | Lac≥10% | 787 (526-849) | 0.067**^a^** |
|  | Lac<10% | 501 (435-692) |  |

a = independent samples t-test and b = Mann-Whitney U-test. State 3 represents active respiration after addition of ADP and State 4 represents the respiration after depletion of ADP. The respiratory control ratio (RCR) was calculated by dividing state 3 by state 4 respiration rate.

**Table S5:** Skeletal muscle dependent mitochondrial respiration in cohort-1.

| **Parameters** | **Group** | **BL** | **BR** | **End** | **P value** |
| --- | --- | --- | --- | --- | --- |
| **Complex 1** |  |  |  |  |  |
| State 3 | Lac≥10% | 1324 (1179-1604) | 1245 (567-1505) | 1105 (924-1503) | 0.800**^a^** |
|  | Lac<10% | 1138 (962-1453) | 1168 (725-1356) | 618 (209-2036) |  |
| State 4 | Lac≥10% | 115 (110-151) | 109 (53-142) | 167 (99-257) | 0.473**^a^** |
|  | Lac<10% | 134 (103-228) | 109 (83-138) | 119 (74-217) |  |
| RCR | Lac≥10% | 10.8 (9.8-13.6) | 8.9 (7.9-11.7) | 8.0 (4.2-11.6) | 0.512**^a^** |
|  | Lac<10% | 8.5 (4.4-14.1) | 10.4 (8.1-11.3) | 7.6 (1.9-10.2) |  |
| **Complex 2** |  |  |  |  |  |
| State 3 | Lac≥10% | 1989 (1747-2645) | 1686 (658-2380) | 2109 (1578-2889) | 0.893**^a^** |
|  | Lac<10% | 2070 (1773-2610) | 1588 (1230-2037) | 1351 (1045-3183) |  |
| State 4 | Lac≥10% | 293 (230-408) | 258 (107-363) | 423 (234-467) | 0.383**^a^** |
|  | Lac<10% | 329 (242-433) | 235 (189-263) | 234 (138-454) |  |
| RCR | Lac≥10% | 7.3 (6.5-8.0) | 6.4 (5.4-7.7) | 6.1 (5.6-6.6) | 0.640**^a^** |
|  | Lac<10% | 6.5 (5.8-8.6) | 7.3 (6.1-7.9) | 6.9 (5.6-7.4) |  |
| **Complex 4** |  |  |  |  |  |
| State 3 | Lac≥10% | 1215 (1001-1660) | 987 (516-1432) | 1431 (1188-2366) | 0.958**^a^** |
|  | Lac<10% | 1393 (837-1626) | 1026 (773-1520) | 1186 (693-3420) |  |

Values represent median (IQR). BL = baseline, BR = immediately before start resuscitation, End = end of the experiment (at 48 hours of resuscitation or before death if earlier), a = time-group interaction with repeated measures analysis of variance including all time points. State 3 represents active respiration after addition of ADP and State 4 represents the respiration after depletion of ADP. The respiratory control ratio (RCR) was calculated by dividing state 3 by state 4 respiration rate.

**Table S6:** Isolated liver mitochondrial respiration in cohort-1.

| **Parameters** | **Group** | **Median (IQR)** | **P value** |
| --- | --- | --- | --- |
| **Complex 1** |  |  |  |
| State 3 | Lac≥10% | 597 (470-895) | 0.405**^a^** |
|  | Lac<10% | 489 (350-956) |  |
| State 4 | Lac≥10% | 169 (117-240) | 0.354**^a^** |
|  | Lac<10% | 131 (88-241) |  |
| RCR | Lac≥10% | 4.3 (3.1-5.2) | 0.917**^a^** |
|  | Lac<10% | 3.6 (2.9-5.4) |  |
| **Complex 2** |  |  |  |
| State 3 | Lac≥10% | 1604 (1383-1968) | 0.093**^b^** |
|  | Lac<10% | 1074 (732-1669) |  |
| State 4 | Lac≥10% | 326 (250-415) | 0.569**^b^** |
|  | Lac<10% | 296 (182-469) |  |
| RCR | Lac≥10% | 4.8 (4.4-6.1) | 0.157**^a^** |
|  | Lac<10% | 4.4 (3.6-5.4) |  |
| **Complex 4** |  |  |  |
| State 3 | Lac≥10% | 743 (624-873) | 0.494**^b^** |
|  | Lac<10% | 589 (412-900) |  |

a = independent samples t-test and b = Mann-Whitney U-test. State 3 represents active respiration after addition of ADP and State 4 represents the respiration after depletion of ADP. The respiratory control ratio (RCR) was calculated by dividing state 3 by state 4 respiration rate.

**Table S7:** Isolated heart mitochondrial respiration in cohort-1.

| **Parameters** | **Group** | **Median (IQR)** | **P value** |
| --- | --- | --- | --- |
| **Complex 1** |  |  |  |
| State 3 | Lac≥10% | 1738 (1324-2033) | 0.623**^a^** |
|  | Lac<10% | 1636 (1097-2268) |  |
| State 4 | Lac≥10% | 293 (261-503) | 1.000**^b^** |
|  | Lac<10% | 421 (217-580) |  |
| RCR | Lac≥10% | 4.5 (3.9-6.4) | 0.551**^a^** |
|  | Lac<10% | 4.9 (3.9-5.8) |  |
| **Complex 2** |  |  |  |
| State 3 | Lac≥10% | 2454 (1912-2893) | 0.408**^a^** |
|  | Lac<10% | 2088 (1965-2708) |  |
| State 4 | Lac≥10% | 744 (594-904) | 0.493**^a^** |
|  | Lac<10% | 861 (561-1198) |  |
| RCR | Lac≥10% | 3.1 (2.9-3.3) | 0.175**^a^** |
|  | Lac<10% | 2.8 (1.7-3.4) |  |
| **Complex 4** |  |  |  |
| State 3 | Lac≥10% | 2319 (1677-2828) | 0.361**^a^** |
|  | Lac<10% | 2262 (1032-2681) |  |

a = independent samples t-test and b = Mann-Whitney U-test. State 3 represents active respiration after addition of ADP and State 4 represents the respiration after depletion of ADP. The respiratory control ratio (RCR) was calculated by dividing state 3 by state 4 respiration rate.

**Table S8.** Composition of study groups in cohort-2.

| Study groups | Lac≥10% | Lac<10% | p value |
| --- | --- | --- | --- |
| n/total n (%) | 7/16 (43.8) | 9/16 (56.2) | 1.000 |
| Low volume | 4/8 (50.0) | 4/8 (50.0) |  |
| High volume | 3/8 (37.5) | 5/8 (62.5) |  |

Values represent n (%). P value with Fisher’s exact test. Lactate clearance was determined as follows: lactate value at 6 hours of resuscitation minus lactate after 12 hours of resuscitation divided by lactate value at 6 hours of resuscitation and the result multiplied by 100.

**Table S9.** Systemic hemodynamics and arterial lactate levels in cohort-2.

| **Parameters** | **Group** | **BL** | **3h** | **6h** | **12h** | **End** | **p value** | **p value^ǂ^**  T*G | **p value^ǂ^**  Time | **p value^ǂ^**  Group |
| --- | --- | --- | --- | --- | --- | --- | --- | --- | --- | --- |
| Heart rate (beats/min) | Lac≥10% | 113 ± 17 | 168 ± 31 | 183 ± 18 | 179 ± 12 | 150 ± 31 | 0.250^a^ | 0.029 | 0.007 | 0.170 |
|  | Lac<10% | 102 ± 9 | 156 ± 31 | 179 ± 28 | 145 ± 41 | 153 ± 49 |  | 0.044^c^ |  |  |
| MAP (mm Hg) | Lac≥10% | 73 ± 12 | 74 ± 14 | 69 ± 11 | 78 ± 10 | 63 ± 26 | 0.033**^a^** | 0.007 | 0.641 | 0.915 |
|  | Lac<10% | 71 ± 25 | 86 ± 23 | 78 ± 25 | 66 ± 28 | 50 ± 20 | 0.279^b^ | 0.312^c^ |  |  |
| MPAP (mm Hg) | Lac≥10% | 16 ± 2 | 25 ± 7 | 22 ± 4 | 22 ± 4 | 29 ± 8 | 0.343^a^ | 0.017 | 0.005 | 0.250 |
|  | Lac<10% | 18 ± 3 | 23 ± 4 | 23 ± 5 | 27 ± 7 | 31 ± 8 |  | 0.124^c^ |  |  |
| CVP (mm Hg) | Lac≥10% | 3 ± 2 | 4 ± 2 | 5 ± 3 | 6 ± 4 | 10 ± 5 | 0.571^a^ | 0.041 | <0.001 | 0.343 |
|  | Lac<10% | 5 ± 2 | 6 ± 3 | 6 ± 3 | 9 ± 3 | 11 ± 4 |  | 0.170^c^ |  |  |
| Cardiac output (ml/kg/min) | Lac≥10% | 87 ± 17 | 104 ± 36 | 104 ± 21 | 90 ± 24 | 132 ± 75 | 0.218^a^ | 0.464 | 0.436 | 0.486 |
|  | Lac<10% | 82 ± 14 | 102 ± 29 | 104 ± 25 | 104 ± 29 | 100 ± 29 |  |  |  |  |
| Arterial lactate (mmol/L) | Lac≥10% | 0.9 ± 0.6 | 1.3 ± 0.4 | 1.5 ± 0.5 | 1.0 ± 2.2 | 1.6 ± 0.5 | 0.007^a^ | 0.002 | 0.145 | 0.984 |
|  | Lac<10% | 0.6 ± 0.2 | 1.2 ± 0.7 | 1.1 ± 0.6 | 1.4 ± 0.7 | 2.6 ± 0.8 |  | 0.168^c^ |  |  |

Values represent mean ± SD. BL = baseline, End = end of experiment after 24 hours of randomization or before death if earlier, MAP = mean arterial blood pressure, MPAP = mean pulmonary artery pressure, CVP = central venous pressure, a = time-group interaction with repeated measures analysis of variance including all time points, b = independent samples t-test at End, ǂ = p values provided with repeated measures analysis of variance including time points 6h and 12h, T*G = time-group interaction, Time = time effect, Group = group effect and c = independent samples t-test at 12h.

**Table S10.** Fractional regional blood flow in cohort-2.

| **Parameters** | **Group** | **BL** | **3h** | **6h** | **12h** | **End** | **p value ^¶^** | **p value^ǂ^**  T*G | **p value^ǂ^**  Time | **p value^ǂ^**  Group |
| --- | --- | --- | --- | --- | --- | --- | --- | --- | --- | --- |
| Carotid artery | Lac≥10% | 5.2 ± 2.3 | 3.8 ± 1.0 | 3.6 ± 0.9 | 3.8 ± 1.3 | 4.5 ± 1.2 | 0.344^a^ | 0.255 | 0.122 | 0.229 |
|  | Lac<10% | 4.9 ± 1.3 | 4.2 ± 1.6 | 3.9 ± 1.1 | 4.9 ± 1.9 | 5.1 ± 1.8 |  |  |  |  |
| Superior mesenteric artery | Lac≥10% | 21.9 ± 9.6 | 16.6 ± 3.7 | 16.7 ± 3.9 | 20.6 ± 6.5 | 21.0 ± 4.3 | 0.379^a^ | 0.474 | 0.021 | 0.421 |
|  | Lac<10% | 16.8 ± 3.3 | 15.8 ± 3.0 | 16.1 ± 1.7 | 18.3 ± 4.0 | 15.8 ± 6.5 |  |  |  |  |
| Celiac trunk | Lac≥10% | 10.8 ± 4.9 | 11.0 ± 3.6 | 9.6 ± 3.7 | 15.0 ± 6.6 | 9.4 ± 4.3 | 0.040^a^ | 0.010 | 0.030 | 0.274 |
|  | Lac<10% | 10.8 ± 4.2 | 8.9 ± 2.8 | 9.9 ± 4.4 | 9.3 ± 5.1 | 7.3 ± 2.3 | 0.242^b^ | 0.075^c^ |  |  |
| Hepatic artery | Lac≥10% | 4.7 ± 3.4 | 5.4 ± 2.8 | 4.9 ± 2.7 | 7.1 ± 3.3 | 4.4 ± 1.7 | 0.018^a^ | 0.015 | 0.068 | 0.050 |
|  | Lac<10% | 4.6 ± 2.2 | 3.0 ± 1.3 | 3.7 ± 1.9 | 3.3 ± 1.8 | 2.6 ± 1.0 | 0.019^b^ | 0.012^c^ |  |  |
| Portal vein | Lac≥10% | 24.0 ± 7.7 | 19.0 ± 4.5 | 18.7 ± 5.6 | 22.2 ± 4.8 | 21.8 ± 6.3 | 0.222^a^ | 0.422 | 0.121 | 0.597 |
|  | Lac<10% | 23.7 ± 6.1 | 22.6 ± 6.5 | 21.6 ± 5.9 | 22.7 ± 9.5 | 18.0 ± 9.9 |  |  |  |  |
| Total hepatic* | Lac≥10% | 26.5 ± 6.5 | 24.0 ± 4.0 | 25.0 ± 4.1 | 25.8 ± 5.44 | 22.4 ± 8.0 | 0.230^a^ | 0.161 | 0.073 | 0.842 |
|  | Lac<10% | 30.4 ± 7.1 | 26.1 ± 7.7 | 24.2 ± 9.0 | 29.1 ± 10.9 | 23.6 ± 10.1 |  |  |  |  |
| Spleen artery | Lac≥10% | 1.4 ± 0.8 | 1.0 ± 0.7 | 1.0 ± 0.8 | 1.3 ± 0.8 | 0.8 ± 0.7 | 0.068^a^ | 0.025 | 0.525 | 0.907 |
|  | Lac<10% | 1.3 ± 0.3 | 1.4 ± 0.8 | 1.3 ± 0.8 | 1.1 ± 0.7 | 0.5 ± 0.2 |  | 0.669^c^ |  |  |
| Renal artery | Lac≥10% | 6.0 ± 1.9 | 5.1 ± 0.8 | 4.4 ± 1.4 | 5.9 ± 2.1 | 3.4 ± 1.4 | 0.021^a^ | 0.018 | 0.968 | 0.635 |
|  | Lac<10% | 7.1 ± 2.1 | 5.3 ± 1.6 | 5.4 ± 2.6 | 4.0 ± 2.1 | 2.2 ± 1.5 | 0.141^b^ | 0.094^c^ |  |  |

Values represent mean ± SD. All fractional blood flows are in %. BL = baseline, End = end of experiment after 24 hours of randomization or before death if earlier, * = sum of hepatic artery and portal vein blood flows, a = time-group interaction with repeated measures analysis of variance including all time points, b = independent samples t-test at End, ǂ = p values provided with repeated measures analysis of variance including time points 6h and 12h, T*G = time-group interaction, Time = time effect, Group = group effect and c = independent samples t-test at 12h.

**Table S11.** Liver tests and hepatic oxygen consumption in cohort-2.

| **Parameters** | **Group** | **BL** | **End** | **p value^a^**  **T*G** | **p value^a^**  **Time** | **p value**  **Group** |
| --- | --- | --- | --- | --- | --- | --- |
| Alanine aminotransferase | Lac≥10% | 17.6 ± 6.7 | 16.0 ± 9.6 | 0.309 | 0.096 | 0.757 |
| (U/L) | Lac<10% | 18.7 ± 9.0 | 12.6 ± 8.4 |  |  |  |
| Aspartate aminotransferase | Lac≥10% | 107.4 ± 45.9 | 140.0 ± 97.1 | 0.286 | 0.413 | 0.327 |
| (U/L) | Lac<10% | 99.9 ± 47.8 | 95.4 ± 44.4 |  |  |  |
| Total bilirubin, median (IQR) | Lac≥10% | 0.0 (0.0-1.0) | 1.0 (0.0-1.0) |  | 0.157**^b^** |  |
| (mg/dL) | Lac<10% | 0.0 (0.0-0.0) | 0.0 (0.0-1.0) |  | 0.083**^b^** |  |
| Hepatic VO_2_, median (IQR) | Lac≥10% | 0.9 (0.7-1.2) | 0.9 (0.9-1.1) |  | 0.655**^b^** |  |
| (ml/kg/min) | Lac<10% | 1.0 (0.8-1.0) | 0.9 (0.8-1.1) |  | 0.893**^b^** |  |

Values represent mean ± SD or median (IQR) when indicated. VO_2_ = oxygen consumption. a = time-group interaction with repeated measures analysis of variance, b = time effect within groups calculated with Wilcoxon Signed Rank Test.

**Figure S1.** Time course of blood lactate levels in cohort-2 animals.


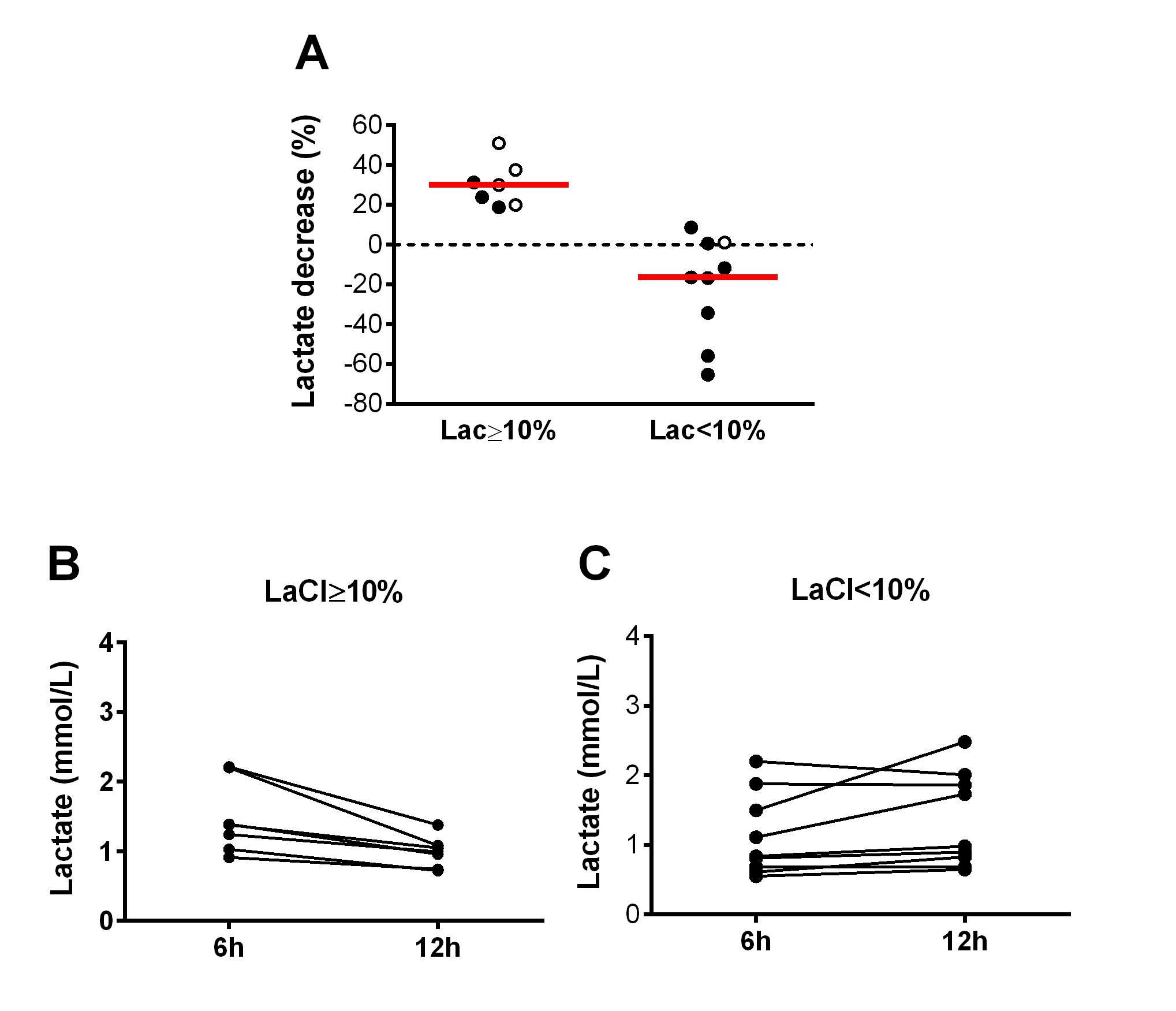


Panel A shows changes in blood lactate levels (%) during the first 6 hours of resuscitation accordingly to study groups. Panels B and C show individual arterial lactate levels after first six hours (6h) and twelve hours (12h) of resuscitation in Lac≥10% and Lac<10% groups. Filled circles in Panel A represent animals that died during the resuscitation period. Red horizontal bars represent median values.

**Figure S2.** Kaplan-Meier curves for 24-hour survival after randomization in cohort-2.

**
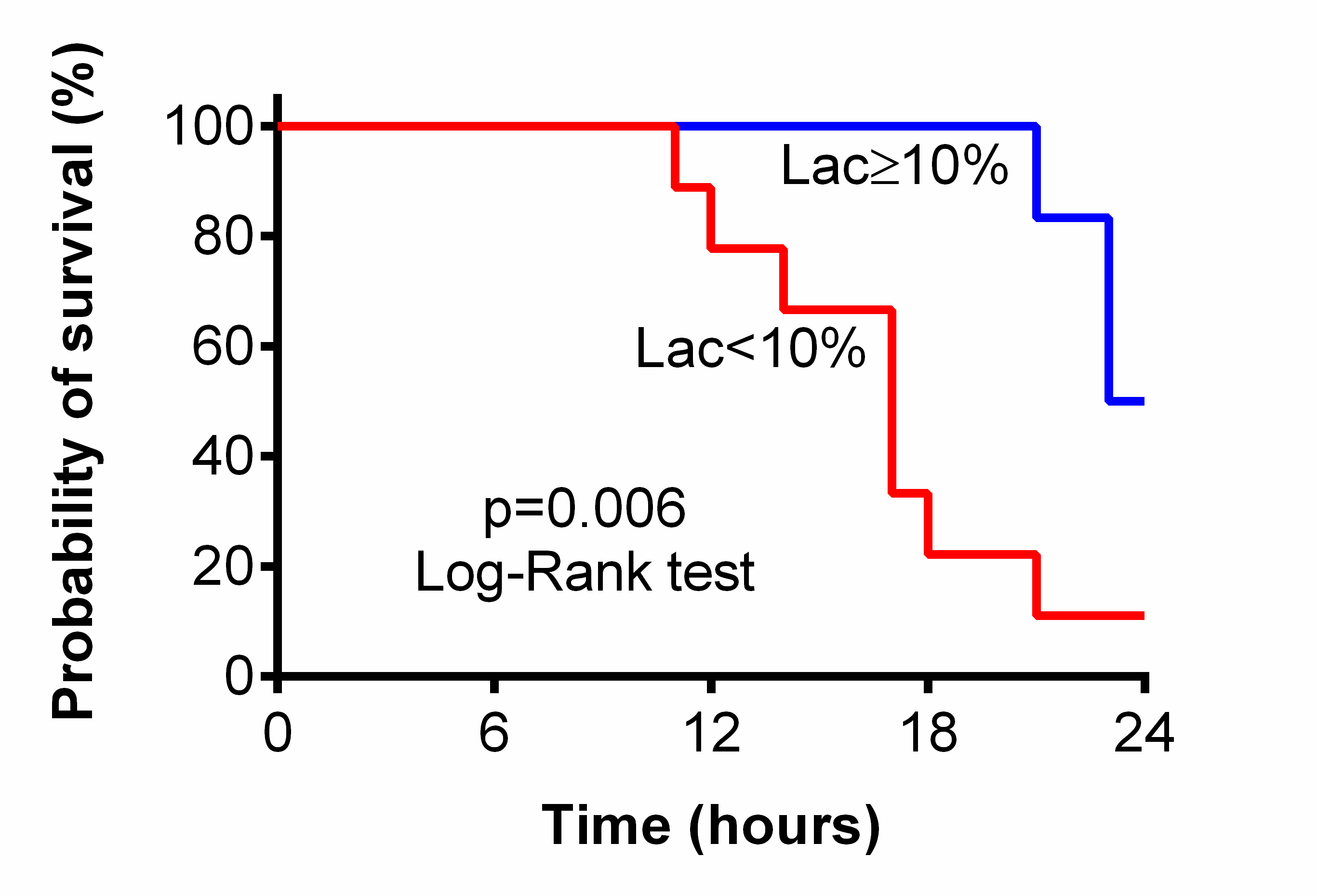
**
